# Supplementary material for: Impact of different heat wave definitions on daily mortality in Bandafassi, Senegal
Source: PLoS One. 2021 Apr 5;16(4):e0249199. doi: 10.1371/journal.pone.0249199 (PMC8021182; doi:10.1371/journal.pone.0249199)
Supplement: S1 Fig — (DOCX) [file pone.0249199.s002.docx]

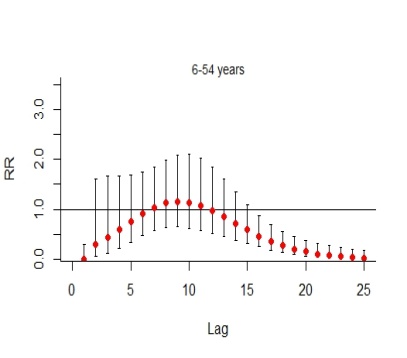

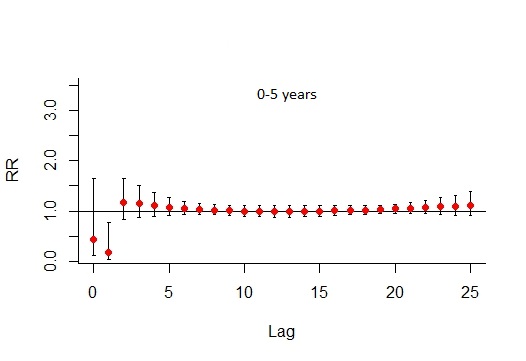

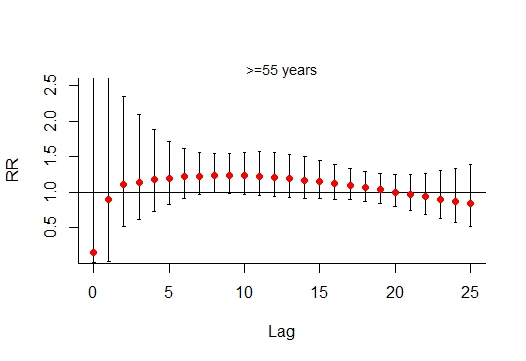


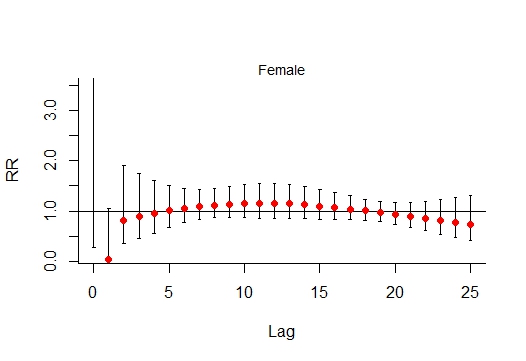

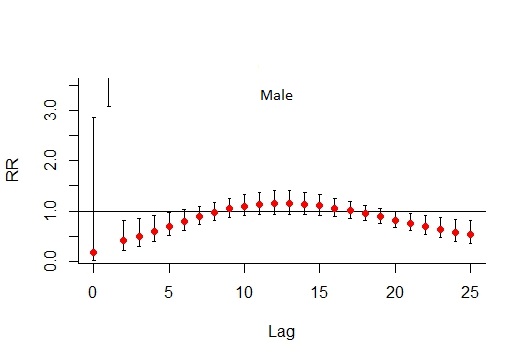

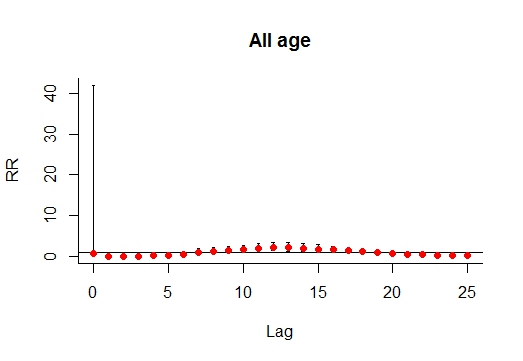


**S1 Fig. Relative Risk (RR) of mortality on the lag distribution of heat wave stratiﬁed by gender, and age based on the deﬁnition ≥ 90th percentile of apparent temperature with duration ≥ 3 consecutive days as heat wave.**
