# Supplementary figures and images for: Impact of different heat wave definitions on daily mortality in Bandafassi, Senegal
Source: PLoS One. 2021 Apr 5;16(4):e0249199. doi: 10.1371/journal.pone.0249199 (PMC8021182; doi:10.1371/journal.pone.0249199)

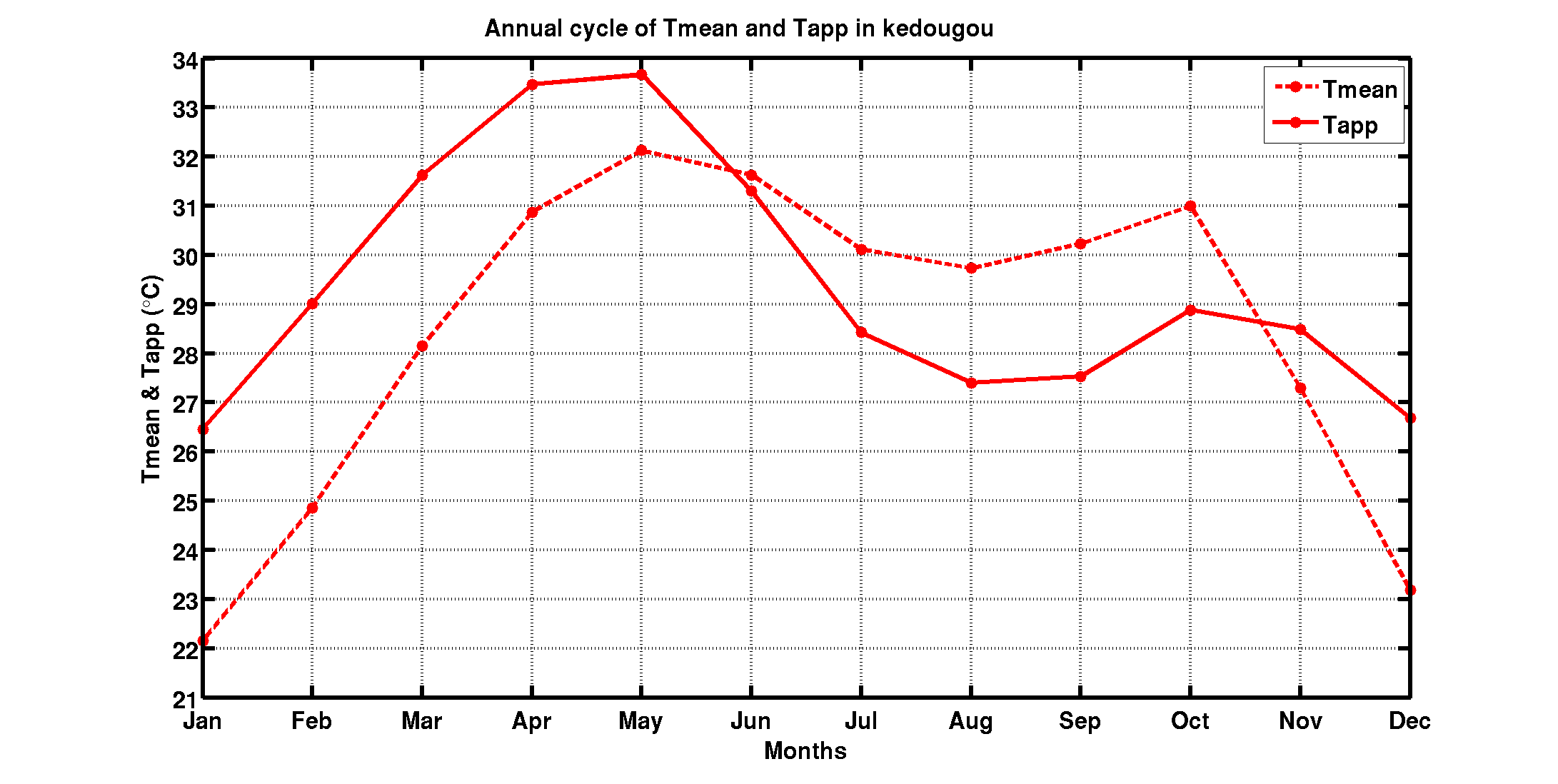


**S3 Fig. Annual cycle of Tmean (mean temperature) and Tapp (mean apparent temperature) in Kedougou (1973-2012).**

Supplement: S3 Fig — (DOCX) [file pone.0249199.s004.docx]
