# Supplementary material for: Impact of different heat wave definitions on daily mortality in Bandafassi, Senegal
Source: PLoS One. 2021 Apr 5;16(4):e0249199. doi: 10.1371/journal.pone.0249199 (PMC8021182; doi:10.1371/journal.pone.0249199)
Supplement: S4 Fig — (DOCX) [file pone.0249199.s005.docx]

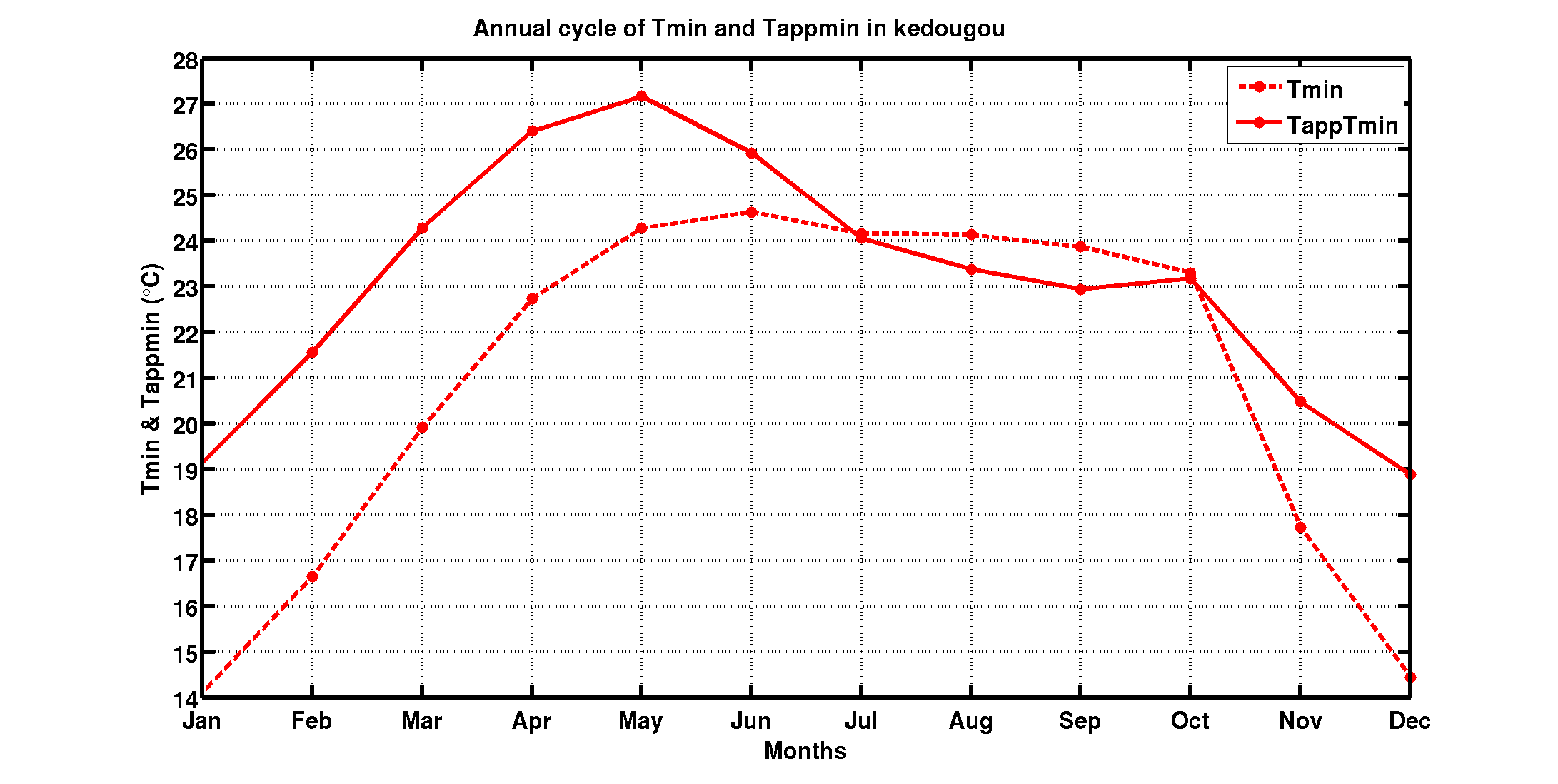


**S4 Fig. Annual cycle of Tmin (minimum temperature) and Tappmin (minimum apparent temperature) in Kedougou (1973-2012).**
