# Supplementary material for: Impact of different heat wave definitions on daily mortality in Bandafassi, Senegal
Source: PLoS One. 2021 Apr 5;16(4):e0249199. doi: 10.1371/journal.pone.0249199 (PMC8021182; doi:10.1371/journal.pone.0249199)
Supplement: S1 Table — Ambient temperature is the best predicteur of mortality in our study in term of AIC because the results with ambient temperature produced the lowest AIC value. (DOCX) [file pone.0249199.s006.docx]

**S1 Table. Displays the comparison (between apparent temperature and ambient temperature) of the sum of AIC values of all gender- and age- speciﬁc mortality for different heat wave deﬁnitions.** Ambient temperature is the best predicteur of mortality in our study in term of AIC because the results with ambient temperature produced the lowest AIC value.

| **Heat wave threshold**  (percentile of temperature) | **Value of AIC** |
| --- | --- |
|  | **3 days 4 days 5 days** |
| **Ambient temperature**  87th 44676.5 44662.3 44664.3  90th **44660.4** 44670.2 44677.2  92th 44670.9 44676.4 44676.0  95th 44675.5 44678 44677.1  97th 44675.8 44676.0 44676.0  **Apparent temperature**  87th 504883.3 50775.6 50891.7  90th **50770.3** 50779.4 50787.2  92th 50821.2 50811.7 50779.6  95th 50818.5 50859.1 50799.4  97th 50899.5 50849.2 50819.6 | |
