# Supplementary material for: Impact of different heat wave definitions on daily mortality in Bandafassi, Senegal
Source: PLoS One. 2021 Apr 5;16(4):e0249199. doi: 10.1371/journal.pone.0249199 (PMC8021182; doi:10.1371/journal.pone.0249199)
Supplement: S2 Table — (DOCX) [file pone.0249199.s007.docx]

**S2 Table.** **Relative Risk (RR) of daily mortality during heat wave based in different duration (≥3, ≥4, ≥5 days) and intensities (87th, 90th, 92th, 95th, 97th percentile of apparent temperature) during the period 1973-2012.**

| **Mortality** | **3 days 4 days 5 days** |
| --- | --- |
|  | **RRs 95%CI RRs 95%CI RRs 95%CI** |
| **Total** | |
| 87th 1.05 (0.9-1.12) 1.2 (1.08-1.25)* 1.08 (0.92-1.5)  90th 1.32 (0.91-1.47)* 1.2 (1.04-1.5) 1.5 (1.07-2.6)  92th 1.17 (0.5-1.9) 1.02 (0.89-1.8) 1.25 (1.23-2.05  95th 2.01 (1.98-3.5) 1.3 (-0.27-2.5) 0.21 (-2.8-3.1)  97th — — — — — — | |
| **Female** | |
| 87th 1.001 (0.65-1.01) 1.03 (1.01-1.41)* 1.24 (1.16-2.09)  90th 1.61 (1.36-1.74)* 1.18 (1.051-2.11) 1.34 (0.17-1.72)  92th 1.22 (1.36-2.03) 1.54 (0.91-1.83) 1.39 (0.9-1.88)  95th 1.54 (1.08-2.8) 0.17 (-2.35-3.52) 0.48 (-1.84-3.1)  97th — — — — — — | |
| **Male** | |
| 87th 1.01 (0.28-1.13) 1.13 (1.01-1.22)* 1.47 (0.16-1.85)  90th 1.05 (0.17-1.67) 1.22 (0.87-1.57) 1.71 (1.17-2.19)  92th 1.18 (1.09-2.16) 1.23 (1.12-2.105) 1.18 (0.98-1.97)  95th 3.8 (2.8-4.8) 4.056 (1.7-6.41) 0.19 (-3.5-4.2)  97th 1.32 (0.07-2.63) — — — — | |
| **0-5 years** | |
| 87th 1.018 (0.9-1.18) 1.11 (1.018-1.25) 1.03 (0.84-1.16)  90th 1.44 (0.13-1.69) 1.2 (0.87-1.53) 1.3 (1.001-1.79)  92th 1.65 (1.45-2.13) 1.36 (0.87-1.77) 1.31 (0.7-1.65)  95th 4.2 (3.2-5.1) 0.56 (-6.5-7.6) — —  97th — — — — — — | |
| **6-54 years** | |
| 87th 0.89 (0.59-1.075) 1.065 (0.88-1.38) 1.35 (0.9-1.69)  90th 1.52 (0.91-1.89) 1.16 (0.91-2.043) 1.82 (1.15-2.18)  92th 1.69 (1.012-2.012) 1.75 (1.15-2.18) 1.44 (0.98-2.11)  95th 1.22 (-0.81-3.09) 2.31 (-0.58-5.83) 0.78 (-3.31-4.21)  97th — — — — — — | |
| ≥**55 years** | |
| 87th 1.08 (0.9-1.87) 1.5 (1.15-1.71)* 1.66 (0.97-1.98)  90th 1.52 (0.89-1.89) 1.44 (1.48-1.92)* 1.96 (1.42-2.12)  92th 1.69 (1.3-2.14) 1.68 (1.25-2.097) 1.84 (0.96-2.47)  95th 2.22 (1.27-3.62) 2.96 (0.84-5.65) — —  97th — — — — — — | |

**---** = not enough data to generate a reliable estimate; ***p-value < 0.001; **p-value < 0.01; *p-value < 0.05.
