# Supplementary material for: Impact of different heat wave definitions on daily mortality in Bandafassi, Senegal
Source: PLoS One. 2021 Apr 5;16(4):e0249199. doi: 10.1371/journal.pone.0249199 (PMC8021182; doi:10.1371/journal.pone.0249199)
Supplement: Attachment — Submitted filename: Reponse to Reviewers.docx [file pone.0249199.s009.docx]

February, 2021

Re: Resubmission of “ Impact of Different Heat Wave Definitions on Daily Mortality in Bandafassi, Senegal”, manuscript id: PONE-D-20-27586R1

Dear Editor :

Thank you for the second round of reviewer’s comments and opportunity to revise our manuscript titled “ Impact of Different Heat Wave Definition on Daily Mortality in Bandafassi, Senegal”. We appreciate the careful reviews and constructive suggestions by the reviewers. The manuscript has substantially improved after making the suggested amendments.

In the following section, find a detailed point-by-point response in red to the reviewers. Changes made in the manuscript are marked using track changes. The revision has been developed in consultation with all co-authors, and each author has given approval to the final version of the manuscript.

Thank you for your consideration.

Sincerely,

Mbaye Faye

On behalf of the authors,

Saint-Louis, Senegal

Reponses to Reviewer #1 comments

I would like to thank the authors for their work in updating the manuscript "Impact of Different Heat Wave Definition on Daily Mortality in Bandafassi, Senegal" based on the 1st round of revision. I recommend minor revision for the 2nd round of the review.

We appreciate the reviewer’s comments for the second round of the review. These comments will help us to improve the quality of the manuscript. For more detail, please refer to the responses below.

**Comment 1**: L124-127: "The full name of the institutional ... this study.". I believe this is not a correct place for this statement. It should be moved to Acknowledgments.

**Answer 1**: Thank you for noting this. However, during the 1st review process, the editor (Orsolya Voros) recommend that us to insert it into the beginning of the Methods section:

‘‘Please insert your ethics statement:

‘‘The full name of the institutional review board that approved our specific study is Bandafassi Health and Demographic Surveillance System (HDSS). We confirm that the Bandafassi Health and Demographic Surveillance System (HDSS) approved this study.’’

into the beginning of the Methods section of your manuscript file.’’

However, we have reworded this text in the revised version of manuscript:

‘‘The full name of the institutional review board that reviewed our specific study approval is the Bandafassi Health and Demographic Surveillance System (HDSS). We confirm that this was approved.’’

**Comment 2**: Figure 2 doesn't look professional. I believe the author should correct the x-axis and make an incremental scale and then highlight the peak days by vertical lines. Also the x-axis label should be moved down and not overlap any other text.

**Answer 2**: We thank the reviewer for raising the issue. As you suggested, we have now corrected the x-axis of Figure 2 in the revised manuscript.

**Comment 3**: I believe the author should consider English proofreading service for this manuscript to improve it.

**Answer 3**: We thank the reviewer for this suggestion. An English proofreading service has been made for the revised manuscript as suggested.

Reponses to Reviewer #2 comments

The authors have addressed all the comments that I made on their manuscript sufficiently. Therefore, I recommend that the manuscript maybe sonsidered.

Thank you for the positive feedback. We appreciate this suggestion which help to improve the readability of our manuscript.

*.*
